# Intestinal Microbiota Signatures Associated with Inflammation History in Mice Experiencing Recurring Colitis

**DOI:** 10.3389/fmicb.2015.01408

**Published:** 2015-12-15

**Authors:** David Berry, Orest Kuzyk, Isabella Rauch, Susanne Heider, Clarissa Schwab, Eva Hainzl, Thomas Decker, Mathias Müller, Birgit Strobl, Christa Schleper, Tim Urich, Michael Wagner, Lukas Kenner, Alexander Loy

**Affiliations:** ^1^Division of Microbial Ecology, Department of Microbiology and Ecosystem Science, Research Network Chemistry meets Microbiology, University of ViennaVienna, Austria; ^2^Department of Microbiology, Immunobiology and Genetics, Max F. Perutz Laboratories, University of ViennaVienna, Austria; ^3^Clinical Institute of Pathology, Medical University of ViennaVienna, Austria; ^4^Division of Archaea Biology and Ecogenomics, Department of Ecogenomics and Systems Biology, University of ViennaVienna, Austria; ^5^Institute of Animal Breeding and Genetics, University of Veterinary Medicine ViennaVienna, Austria; ^6^Ludwig Boltzmann Institute for Cancer ResearchVienna, Austria; ^7^Department of Laboratory Animal Pathology, University of Veterinary Medicine ViennaVienna, Austria

**Keywords:** DSS, colitis, IBD, FISH, *Mucispirillum*, *Akkermansia*, *Bacteroides*

## Abstract

Acute colitis causes alterations in the intestinal microbiota, but the microbiota is thought to recover after such events. Extreme microbiota alterations are characteristic of human chronic inflammatory bowel diseases, although alterations reported in different studies are divergent and sometimes even contradictory. To better understand the impact of periodic disturbances on the intestinal microbiota and its compositional difference between acute and relapsing colitis, we investigated the beginnings of recurrent inflammation using the dextran sodium sulfate (DSS) mouse model of chemically induced colitis. Using bacterial 16S rRNA gene-targeted pyrosequencing as well as quantitative fluorescence *in situ* hybridization, we profiled the intestinal and stool microbiota of mice over the course of three rounds of DSS-induced colitis and recovery. We found that characteristic inflammation-associated microbiota could be detected in recovery-phase mice. Successive inflammation episodes further drove the microbiota into an increasingly altered composition post-inflammation, and signatures of colitis history were detectable in the microbiota more sensitively than by pathology analysis. Bacterial indicators of murine colitis history were identified in intestinal and stool samples, with a high degree of consistency between both sample types. Stool may therefore be a promising non-invasive source of bacterial biomarkers that are highly sensitive to inflammation state and history.

## Introduction

The intestinal microbiota is increasingly acknowledged to play an important role in health and disease (Clemente et al., 2012; Flint et al., 2012; Tremaroli and Backhed, 2012; Cotillard et al., 2013). Long-term studies have demonstrated that the adult human and mouse microbiota retains a relatively similar composition over months and even years (Caporaso et al., 2011; Schloss et al., 2012; Faith et al., 2013; David et al., 2014a). However, microbial communities in all ecosystems are subject to disturbances; changes in conditions that directly or indirectly alter the community (Shade et al., 2012). The intestinal microbiota is no exception. For example, shifts in diet can produce rapid and pronounced alterations in the microbiota (David et al., 2014a,b). In addition to diet, stressors such as antibiotic treatment, enteric infection, and colitis can induce remodeling of the gut microbiota (Garrett et al., 2007; Stecher et al., 2010; Dethlefsen and Relman, 2011; Lepage et al., 2011).

The insensitivity of a community to disturbance is referred to as its stability. Stability can be measured both in terms of resistance, the degree to which a community is unresponsive to a change in conditions, as well as the resilience, the tendency for a community to return to its original state following a disturbance (Shade et al., 2012). For example, long-term dietary patterns promote a gut microbiota structure that is largely resistant to short term dietary changes (Wu et al., 2011). Likewise, shifts in the gut microbiota driven by dramatic changes in diet are reversible upon reversion to the original diet (David et al., 2014b). Therefore in the face of dietary disturbances the gut microbiota of healthy adults can be characterized as relatively stable, both in terms of resistance and resilience. Considering stressors, a single treatment with antibiotics (amoxicillin or ciprofloxacin) has been shown to induce major changes in the gut microbiota (De La Cochetière et al., 2005; Dethlefsen et al., 2008; Dethlefsen and Relman, 2011). Though the pre-antibiotic microbiota was largely restored after treatment, some taxa were lost and replaced by genetically similar (at the 16S rRNA gene level) organisms. Organism replacement in antibiotic treatment seems to partially involve functional redundancy and stochastic shuﬄing of members of the microbiota, but functional shifts have also been observed (Pérez-Cobas et al., 2013). Interestingly, the microbiota response to multiple ciprofloxacin treatments was distinct, suggesting that the history of antibiotic treatments has an effect on the microbiota (Dethlefsen and Relman, 2011).

Inflammatory bowel disease (IBD), which includes Crohn’s disease and ulcerative colitis, are increasingly prevalent multifactorial diseases in which the microbiota is thought to play a role (Berry and Reinisch, 2013). IBD is characterized by chronic and relapsing intestinal inflammation (Huttenhower et al., 2014). Active inflammation of the human gut is associated with dramatic changes in the microbiota, but results from published studies of IBD are characterized by extensive inter-individual and inter-study variability, leading to divergent or even contradictory results among multiple studies (Berry and Reinisch, 2013; Huttenhower et al., 2014). This is in stark contrast to animal models of acute inflammation in which microbiota responses are generally highly reproducible (Nagalingam et al., 2011; Berry et al., 2012; Liang et al., 2014; Rauch et al., 2014; Schwab et al., 2014; Ritchie et al., 2015). Divergent reports in IBD may be at least partially due to differences in disease development and inflammation history. To systematically address the difference between acute and relapsing colitis on the microbiota, we characterized the temporal stability of the intestinal microbiota in inflammation-naïve mice as well as mice with a history of repeated inflammation episodes using the DSS model of chemically induced colitis. We observed that although early recovery-phase microbiota shared reproducible similarities across repeated colitis events, the number of previous colitis episodes affected microbiota composition. These data illustrate that the gut microbiota is largely resilient to inflammation-induced conditions, but also that colitis history leaves an imprint on the early recovery-phase microbiota even after only a single colitis event.

## Materials and Methods

### Animal Experiments

C57BL/6 mice 6–8 weeks of age at the start of the experiment were used. All replicates were performed simultaneously and the animals were ordered at the same time from the same animal facility. The experiment consisted of three 19 day cycles of treatment and recovery, with a total of 11 mice in treatment group and eight mice in control group. For each treatment cycle animals were provided 2% dextran sodium sulfate (DSS, molecular weight: 36–50 kDa, MP Biomedicals) in autoclaved drinking water *ad libitum* for 5 days. Treated mice were randomly assigned to cages, but cage did not appreciably affect microbiota composition [permutational multivariate analysis of variance (perMANOVA), *p* = 0.9]. From three to five mice were sacrificed at the start of the experiment and in the recovery phase after the conclusion of DSS treatment for full intestinal flush samples. Additionally, fecal pellets from eight mice (five DSS-treated and three untreated controls) were collected at multiple time points throughout the experiment. Conclusion of the recovery phase was based on regain of body weight to the level of control animals. Upon sacrifice the cecum and colon were immediately removed and flushed with 7 ml of sterile phosphate-buffered saline (PBS; 136 mM NaCl, 2.6 mM KCl, 10 mM Na_2_HPO_4_, 1.7 mM KH_2_PO_4_, pH 7.2) as described previously (Berry et al., 2012). Pooled cecum and colon samples were used to generate a comprehensive profile of the microbiota composition in the lower intestinal tract and has been previously validated for DSS treatment studies (Berry et al., 2012). The intestine was prepared as a Swiss roll, fixed in 4% paraformaldehyde, embedded in paraffin, and sectioned for pathology evaluation as described previously (Stevceva et al., 1999; Williams et al., 2001; Pflegerl et al., 2009). Flushed cecum and colon contents were homogenized, collected by centrifugation and snap-frozen for DNA purification. All animal experiments were discussed and approved by the institutional ethics committee and conducted in accordance with protocols approved by the Austrian laws (BMWF-66.006/0002-II/10b/2010).

### DNA Purification and 16S rRNA Gene Amplicon Pyrosequencing

Nucleic acids were extracted with a standard protocol employing phenol–chloroform and bead-beating (Griffiths et al., 2000). DNA quality and quantity was assessed with agarose gel electrophoresis and spectrophotometry (NanoDrop 1000, Thermo Scientific). PCR primers targeting a fragment of the 16S rRNA gene (V6–V9 region) of most *Bacteria* were used (909F, 5′-ACTCAAAKGAATWGACGG-3′ and 1492R, 5′-NTACCTTGTTACGACT-3′; Berry et al., 2011). The pyrosequencing primers included the template-specific sequence, the sequencing primer and an 8 nt barcode (Hamady et al., 2008). Amplicon libraries were produced from DNA of both pooled cecum and colon contents and freshly collected fecal pellets. A two-step, low cycle number PCR protocol was used to amplify template DNA to minimize bias associated with barcoded pyrosequencing primers (Berry et al., 2011). PCR amplicons from triplicate amplifications were pooled and purified using Agencourt AMPure beads (Beckman Coulter Genomics) and quantified with a fluorescent-stain-based kit (Quant-iT PicoGreen, Invitrogen). Pyrosequencing was performed with Titanium reagents on a 454 genome sequencer FLX (Roche) as recommended by the manufacturer. All pyrosequencing data in the study are archived at NCBI Sequence Read Archive (SRA) under Accession SRP008057.

### Sequencing Data Analysis

Reads were quality-filtered using the amplicon pipeline of the GS Run Processor (Roche) and the PyroNoise algorithm in Mothur (Schloss et al., 2011). Sequencing reads were de-multiplexed using QIIME (Caporaso et al., 2010) and clustered with UCHIME (Edgar et al., 2011) into operational taxonomic units (OTUs) at 97% identity (with a minimum length of 250 nt). Taxonomic classification was made using the Ribosomal Database Project (RDP) naïve Bayesian classifier using the Greengenes database (February 4, 2011 release; Wang et al., 2007). Alpha and beta diversity metrics, analysis of similarity (ANOSIM), non-parametric perMANOVA, and redundancy analysis were calculated using the vegan package (Oksanen et al., 2013). Indicator species analysis was performed using the indicspecies package in R (De Cáceres and Legendre, 2009) and correction for multiple testing was performed with the FDR method in R. Analysis of variance and Tukey *post hoc* test were also performed in R. A tree was constructed from near-full-length 16S rRNA reference gene sequences closely related to sequences using the maximum likelihood method RAxML (Stamatakis et al., 2008). Bootstrap analysis was performed (rapid bootstrap algorithm) using 500 bootstrap iterations. OTU sequences were then added to the tree using the quick-add parsimony method in ARB (Ludwig et al., 2004).

### Fluorescence *In Situ* Hybridization

For fluorescence *in situ* hybridization (FISH) analysis, oligonucleotide probes specific for the 16S rRNA or 23S rRNA of target organisms were hybridized as described previously (Berry et al., 2012) using formamide concentrations optimized for each probe for stringent hybridizations (Supplementary Table S1). Negative control probes (complementary to EUB338 I) labeled with the dyes FLUOS, Cy3 and Cy5 were also hybridized to ensure that there was no non-specific binding of probes to the samples. Hybridized samples were imaged on a confocal scanning laser microscope (Zeiss 510 Meta, Oberkochen, Germany). For each quantification, at least 20 fields of view (63X) from each sample were analyzed with the image analysis software daime (Daims et al., 2006).

## Results and Discussion

### Repeated DSS-Induced Colitis

**FIGURE 1:**
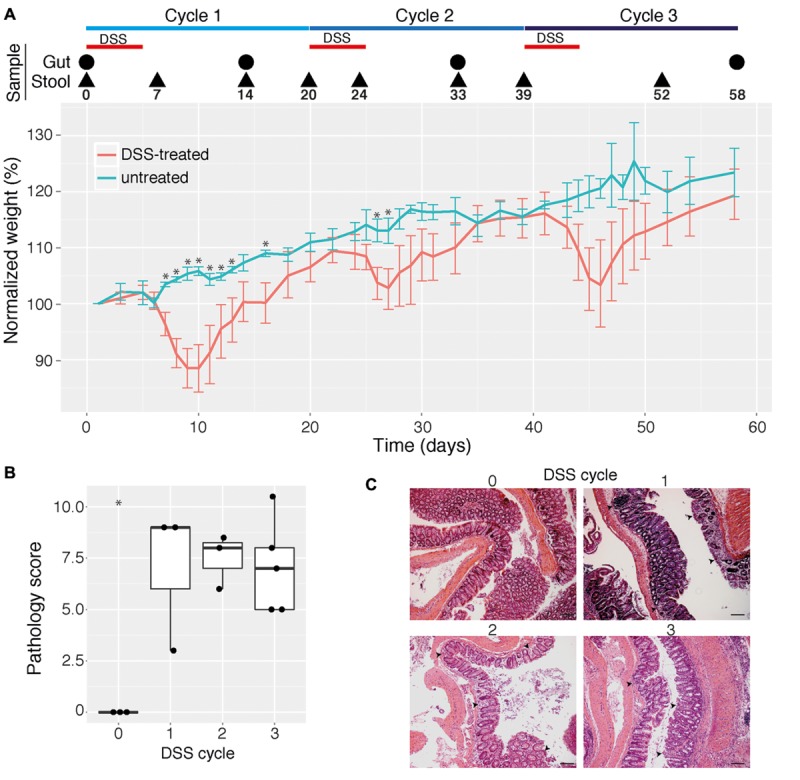
**Effect of multiple cycles of dextran sodium sulfate (DSS) treatment in mice. (A)** Mean change in weight (in percent) for mice given three cycles of 2% DSS in drinking water (indicated by red horizontal bars) as well as untreated control mice (*n* = 3–11 for each data point). Mice were sampled for intestinal (cecum and colon) contents and pathology analysis as well as for stool analysis at selected time points (indicated by a circle or triangle, respectively, and the day number). Error bars indicate 95% confidence interval and asterisks indicate days where for which there is a statistically significant difference in weights between control and treatment groups (*p* < 0.05, corrected for multiple comparisons). Weight dynamics for individual mice are shown in Supplementary Figure S1. **(B)** Pathology scores of colon mucosal tissue from mice give 0, 1, 2, or 3 cycles of DSS. Pathology scores were calculated as the sum of inflammation severity and extent, crypt depth, and area of inflammation, as determined by an experienced pathologist. Asterisk indicates statistically significant difference compared with all other groups (*p* < 0.05). **(C)** Representative photomicrographs (10× magnification) of hematoxylin and eosin-stained colon tissues obtained from untreated and DSS-treated mice. Black scale bars are 200 μm. Arrows indicate infiltrates of immune cells.

Over the course of the experiment mice were treated with DSS to induce colitis (treatment phase) and then allowed to recover from DSS treatment (recovery phase) three times. Acute inflammation induced transient loss of body weight after each DSS treatment (**Figure 1A**, Repeated measures ANOVA, *p* = 0.0017), which is in agreement with previous reports (Berry et al., 2012; Rauch et al., 2014). The inter-mouse variability in weight loss within the treatment group was not significantly different with increasing cycles of DSS treatment (*F* test, *p* > 0.05). The lack of significant differences in weights of treatment and control mice in the third cycle are therefore likely due to lower statistical power arising from reduced numbers of replicates later in the experiment after sacrifice of mice at earlier time points, though may alternatively reflect an adaptation mechanism by the animals in response to repeated inflammation episodes (**Figure 1A**, Supplementary Figure S1). Mouse mucosal tissue sampled at the end of each recovery phase (**Figure 1A**, time points indicated by large black circles) indicated remaining pathological damage (**Figures 1B,C**, ANOVA, *p* = 0.001). Importantly, however, no significant differences in the severity of damage with the number of cycles of DSS treatment were observed (**Figure 1B**, ANOVA, *p* = 0.45), indicating that the three cycles of DSS treatment did not cause appreciable increases in sustained disease pathology.

### Microbiota Signatures of Prior Colitis Episodes in Intestinal Flush Samples

**FIGURE 2:**
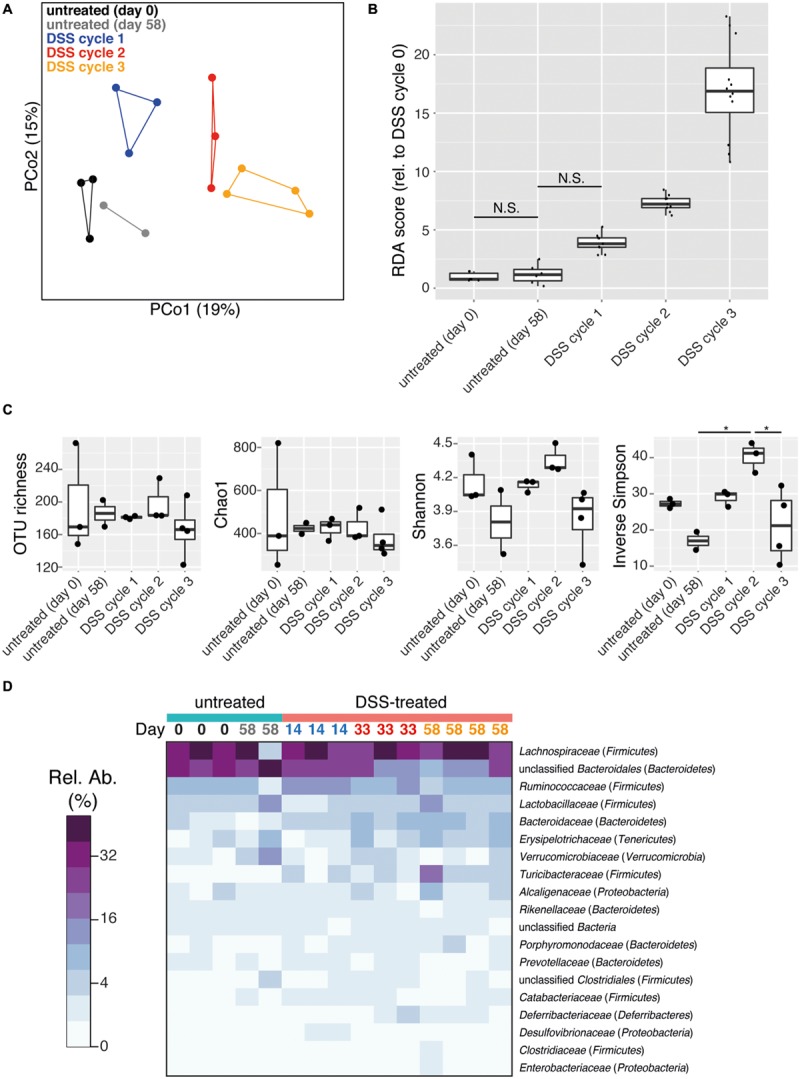
**DSS treatment shapes recovery-phase intestinal microbiota composition and diversity.** All data is from intestinal (cecum and colon) flush samples. **(A)** Principal coordinates analysis (PCoA) using the Bray–Curtis dissimilarity metric reveals clustering of samples based on number of DSS treatment cycles (points are connected by lines to illustrate clustering). This clustering is statistically supported by the ANOSIM test (*R* = 0.3379, *p* = 0.003). **(B)** Differences in microbiota composition relative to day 0 untreated samples based on redundancy analysis are shown. All pairwise comparisons between groups are significant (*p* < 0.05) except for those joined by a horizontal bar labeled N.S. **(C)** Estimation of microbial community richness (observed OTU richness and Chao1 estimated richness) and diversity (Shannon and inverse Simpson indices) for each sample. For all analyses sequence libraries were sub-sampled to 1000 reads. An asterisk indicates a significant difference between groups (ANOVA and Tukey’s HSD *post hoc* test, *p* < 0.05). **(D)** Heatmap of the relative abundance of family-level taxa (rows) for each sample (columns). The abundance is shown in relative percent and the scale is square root transformed to allow visualization of less abundant groups. Families with at least 1% relative abundance in at least one sample are shown. Columns are labeled with sample day and color-coded by DSS cycle (1 = blue, 2 = red, 3 = orange).

The intestinal microbiota is a potential source of non-invasive biomarkers for monitoring health state (Berry and Reinisch, 2013). We were therefore interested in whether the composition of the intestinal microbiota is a more sensitive indicator of inflammation history than mucosal tissue pathology during early recovery phase. We applied a non-parametric multivariate analysis of variance test (perMANOVA) to determine if the microbiota composition of intestinal (cecum and colon) flush samples was associated with previous experimental treatments. This analysis revealed that prior DSS treatment was associated with an altered intestinal microbiota composition (perMANOVA, *p* < 0.001). This result is consistent with a previous report that the intestinal microbiota of mice recovering from DSS treatment has an altered composition (Schwab et al., 2014). Significantly, the number of previous colitis episodes was also an important factor associated with microbiota composition (perMANOVA, *p* = 0.033). These results indicate that despite similar pathology, intestinal microbiota provided a record of the history of inflammation episodes. Consistent with this result, clear clustering of samples by treatment was observed with principal coordinates analysis (PCoA) produced with the Bray–Curtis dissimilarity metric (**Figure 2A**). This clustering was confirmed with the ANOSIM test, which tests the statistical significance of community composition overlap within and between groups (ANOSIM, *R* = 0.3379, *p* = 0.003). This result is consistent with the observation that repeated disturbances by ciprofloxacin treatment affects the gut microbiota differently between the first and second course of antibiotics (Dethlefsen and Relman, 2011). Additionally, increasing cycles of DSS treatment led to an increasingly divergent microbiota from control samples, as determined by redundancy analysis (**Figure 2B**). Though some differences were observed, microbiota richness and alpha diversity, as quantified by multiple commonly used estimators, failed to reveal any consistent trend with respect to treatment (**Figure 2C**). Therefore the treatment-associated alterations in the intestinal microbial community were due to compositional changes rather than reduction in microbial richness or dominance of particular organisms.

The intestinal microbiota was dominated by the phyla *Firmicutes* and *Bacteroidetes*, but *Tenericutes, Verrucomicrobia, Proteobacteria*, and *Deferribacteres* were also present at >1% relative abundance in at least one sample (Supplementary Figure S2). Prior DSS treatment was associated with significant shifts in the microbiota composition at the family level but not the phylum level (perMANOVA, *p* = 0.012 and *p* = 0.076, respectively) in the recovery phase. A dramatic divergent shift was observed within the phylum *Bacteroidetes*, in which an increase in *Bacteroidaceae* (untreated: 1.8 ± 0.05%, DSS: 5.2 ± 0.7%; mean ± SEM) was accompanied by a decrease in unclassified *Bacteroidales* (untreated: 34.7 ± 3.7%, DSS: 19.4 ± 2.6%; mean ± SEM; **Figure 2D**, ANOVA, *p* < 0.005 and *p* < 0.006, respectively). This pattern has been previously associated with acute-phase colitis (Berry et al., 2012; Schwab et al., 2014). Characteristic inflammation-associated microbiota can therefore also be detected even in recovery-phase mice.

### Microbiota Signatures of Prior Colitis Episodes in Fecal Pellet Samples

**FIGURE 3:**
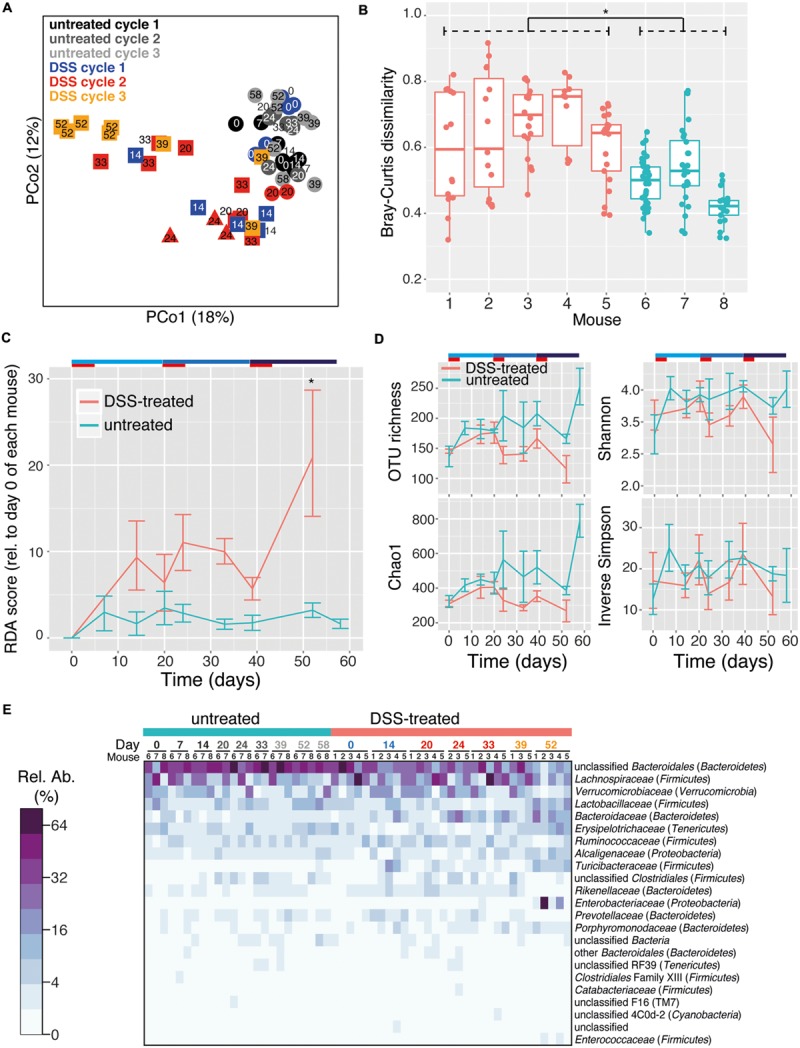
**DSS treatment shapes fecal pellet intestinal microbiota composition and diversity.** Fecal pellets were collected immediately upon defecation from untreated as well as DSS treated (in both acute and recovery phases) mice. **(A)** PCoA using the Bray–Curtis dissimilarity metric displays a trend toward clustering of samples based on number of DSS treatment cycles. This is statistically supported by the ANOSIM test (*R* = 0.3254, *p* = 0.001). Circles indicate untreated mice, triangles and squares indicate the acute and recovery phases of inflammation, respectively. **(B)** Microbiota stability per mouse, as determined by pairwise Bray–Curtis dissimilarity values calculated from multiple samples from each mouse. DSS-treated mice (red, #1–5) had significantly higher dissimilarity values as untreated mice (blue, #6–8), indicating a less stable microbiota composition throughout the duration of the entire experiment (Asterisk indicates *p* < 0.05). **(C)** The mean and standard deviation of the microbial community at successive time points relative to day 0 for each mouse based on redundancy analysis. Higher values indicate increasing divergence from the starting community. An asterisk signifies a difference between treated and untreated mice at that time point (*p* < 0.05). **(D)** Estimation of microbial community richness (observed OTU richness and Chao1 estimated richness) and diversity (Shannon and inverse Simpson indices) for each sample (mean and standard deviation shown). Observed OTU richness and Chao1 estimated richness were significantly reduced in the DSS treated group (ANOVA, *p* < 0.001). For all analyses sequence libraries were sub-sampled to 1000 reads. **(E)** Heatmap of the relative abundance of family-level taxa (rows) for each sample (columns). The abundance is shown in relative percent and the scale is square root transformed to allow visualization of less abundant groups. Families with at least 1% relative abundance in at least one sample are shown.

In order to monitor longitudinal changes in the microbiota of individual mice, fecal microbiota samples from mice undergoing three DSS treatment and recovery cycles were analyzed. Fecal samples therefore included both acute inflammation phase as well as recovery phase (**Figure 1A**). Consistent with lumen flush samples, DSS treatment and treatment cycle were significantly associated with fecal microbiota composition (perMANOVA, *p* < 0.001 for both). PCoA revealed a trend of clustering of fecal pellet microbiota by treatment and DSS cycle (**Figure 3A**, ANOSIM, *R* = 0.3254, *p* = 0.001). Early-phase recovery samples from each cycle (days 14 and 20 for cycle 1 and days 33 and 39 for cycle 2) tended to cluster together. DSS treatment destabilized the fecal microbiota, with community composition varying more greatly in treated mice compared to untreated mice (**Figure 3B**, ANOVA, *p* < 0.001). This result is in line with observations that the microbiota is less stable due to colitis, both for acute as well as relapsing forms such as those characteristic of human IBDs (Morgan et al., 2012; Angelberger et al., 2013; Berry and Reinisch, 2013; Schwab et al., 2014). Interestingly, the microbiota instability caused by multiple episodes of DSS induced-colitis produced a directional change of the microbial community away from the starting community over the course of the experiment, a trend that was not observed in untreated control samples (**Figure 3C** and Supplementary Figure S3, ANOVA, *p* < 0.001). The microbiota is therefore not entirely resilient to repeated acute colitis events, at least in the early recovery phase. This is similar to what has been observed for other stressors such as treatment with antibiotics (De La Cochetière et al., 2005; Dethlefsen and Relman, 2011; Fouhy et al., 2012). This incomplete compositional resilience (Allison and Martiny, 2008) has been suggested to be indicative of a shift of the microbiota to an alternative stable state, which is defined as when a community moves from one relatively stable composition to another stable composition following a disturbance (Shade et al., 2012). In the present study, microbiota resilience was further deteriorated after multiple colitis events. The microbiota may therefore shift into alternative stable states, which include “dysbiotic states” such as those observed in chronic inflammatory diseases in humans (Frank et al., 2007; Morgan et al., 2012). Long-term studies are needed to address how stable these states are over extended periods and if signatures of colitis history are maintained or lost in the absence of additional colitis events. Markedly, the richness of the fecal pellet microbiota decreased with prior DSS treatment and suffered additional reductions with multiple treatments (**Figure 3D**, ANOVA, *p* < 0.001 for both observed OTU richness and Chao1 estimated richness). Though intestinal flush and fecal samples produced largely similar results, a significant reduction in diversity was not observed in intestinal flush samples. This is perhaps due to the large contribution of the cecal microbiota in flush samples, which may not experience as dramatic diversity-altering changes as the colon microbiota during colitis. Ecological theory suggests that environments with intermediate levels of disturbance can support a larger diversity of organisms (Hall et al., 2012). The loss of diversity observed in the present study suggests that the disturbances caused by the DSS stressor are likely far more intense than a diversity-increasing disturbance might be. Further work is needed to determine if the loss of compositional resilience and decrease in OTU richness hold consequences for intestinal microbiota function. It is very well possible that compositional properties give insight into functional properties as, for example, loss of gene diversity has been associated with IBD as well as metabolic disorders and liver cirrhosis (Le Chatelier et al., 2013; Qin et al., 2010, 2014) and is predictive for how successful dietary intervention is on reducing markers of inflammation (Cotillard et al., 2013).

The phylum-level composition of the fecal microbiota was largely similar to the intestinal flush samples, with a predominance of *Bacteroidetes* and *Firmicutes* as well as *Verrucomicrobia, Tenericutes, Proteobacteria*, TM7, and *Cyanobacteria* (Supplementary Figure S4). The phylum-level composition was mouse-specific but was not significantly altered by DSS treatment or treatment cycle (perMANOVA, *p* = 0.015, 0.07, and 0.41, respectively). The family-level composition was also mouse-specific, but was also significantly altered by DSS treatment and treatment cycle (**Figure 3E**, perMANOVA, *p* = 0.02, 0.001, and 0.005, respectively). This shift was characterized by a large decrease in the unclassified *Bacteroidales* (untreated: 45.3 ± 2.6%, DSS: 28.5 ± 2.8%; mean ± SEM) as well as the *Clostridiales* family XIII (untreated: 0.4 ± 0.07%, DSS: 0.2 ± 0.02%; mean ± SEM). In addition, several families increased: *Bacteroidaceae* (untreated: 1.9 ± 0.3%, DSS: 7.8 ± 1.3%; mean ± SEM), *Rikenellaceae* (untreated: 1.1 ± 0.1%, DSS: 2.0 ± 0.3%; mean ± SEM), *Porphyromonadaceae* (untreated: 0.6 ± 0.1%, DSS: 1.6 ± 0.3%; mean ± SEM), and *Turicibacteraceae* (untreated: 0.1 ± 0.05%, DSS: 3.3 ± 0.9%; mean ± SEM). Colitis episodes therefore have a lasting impact on the gut microbiome, and successive episodes further drive the microbiome into an altered composition even after recovery from acute inflammation. It is not possible to conclude from the current study, however, whether complete recovery was achieved between inflammation episodes. Further studies are needed to evaluate whether incomplete or complete recovery between multiple inflammation episodes would have a different impact on the gut microbiota.

### Species-Level Operational Taxonomic Units (OTUs) Associated with Colitis History

**FIGURE 4:**
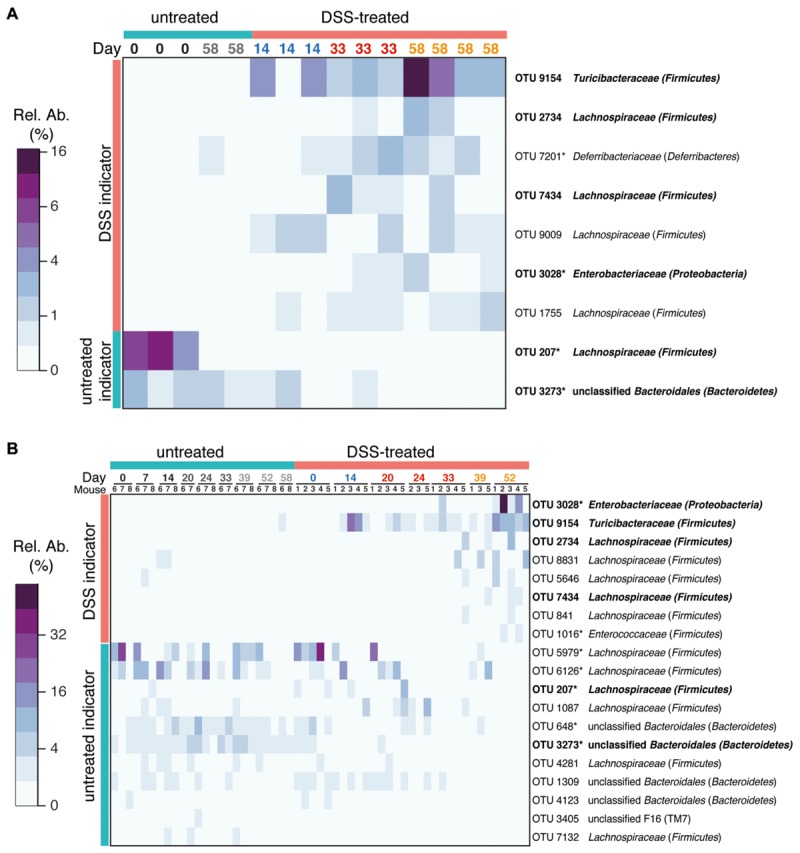
**Bacterial indicators of colitis history.** OTUs characteristic for prior DSS treatment were determined using indicator species analysis. Heatmaps of the relative abundance of each OTU is shown. Only abundant OTUs (>1% relative abundance in at least one sample) were evaluated. **(A)** Indicator OTUs from gut flush samples. **(B)** Indicator OTUs from stool samples. Indicators from stool were identified by comparing samples from untreated mice with samples from mice treated with three cycles of DSS (day 52) using indicator species analysis. Only OTUs that were significantly associated with prior treatment or controls are shown (*p* < 0.05). OTUs identified in both intestinal flush and stool samples are shown in bold. Asterisks after the OTU ID indicates that this OTU was also identified in intestinal flush (cecum and colon) samples as an indicator of acute DSS-induced colitis or of untreated mice in a previous study (all OTUs share 100% sequence similarity over at least 400 nt with previously identified OTUs; Berry et al., 2012). See Supplementary Figure S5 for a phylogenetic tree of indicator OTUs.

We applied an indicator species analysis to identify which OTUs were associated with prior colitis episodes. In order to focus on major members of the gut microbiota only OTUs that had at least 1% relative abundance in one sample were analyzed. In intestinal flush samples, this analysis identified seven OTUs increased in samples from mice with prior DSS-induced colitis (either 1, 2, or 3 cycles of DSS) and two OTUs associated with untreated animals (**Figure 4A**). Indicator OTUs for prior colitis were affiliated with the families *Turicibacteraceae, Enterobacteriaceae, Deferribacteriaceae*, and *Lachnospiraceae*. OTUs associated with untreated samples were associated with *Lachnospiraceae* and unclassified *Bacteroidales*. *Enterobacteriaceae* are often reported to be associated with inflammation and are overrepresented in individuals with IBD (Morgan et al., 2012; Berry and Reinisch, 2013). *Deferribacteriaceae*, which are represented by the genus *Mucispirillum*, are increased during inflammation and have been suggested to be mucus-dwelling commensals that can cause disease, so called-pathobionts, because under some conditions the immune system mounts an IgG response against them (Robertson et al., 2005; Berry et al., 2012; El Aidy et al., 2014). Interestingly, though no shifts were observed in the family *Lachnospiraceae* as a whole, members of this family have divergent resilience to colitis events. This mirrors the extensive diversity of responses to acute colitis by members of this family (Berry et al., 2012) and suggests the presence of unidentified physiological differences in this group responsible for varying competitiveness during and in the wake of colitis.

Indicator species analysis of stool samples identified 19 indicators (eight for three cycles of DSS treatment and 11 for untreated controls, **Figure 4B**, Supplementary Figure S5). The majority of indicators from intestinal flush samples were also identified as indicators in stool samples (six of nine). Interestingly, outgrowth of some indicators, such as *Turicibacteraceae* OTU 9164, occur to the same extent in each recovery phase, whereas others such as *Enterobacteriaceae* OTU 3028 and some OTUs classified as *Lachnospiraceae* are increased to a greater extent in the recovery phase of later DSS cycles. This may reflect a subtle difference in the fitness of these taxa with increasing number of inflammation episodes, but little is known about the physiology of members of the *Turicibacteraceae* and how they differ from *Enterobacteriaceae* and *Lachnospiraceae*. All three groups likely have an advantage in disturbed gut environment, either by an altered nutrient environment during inflammation, as has been shown for *Escherichia coli* (Winter et al., 2013), or by a loss of colonization resistance after acute inflammation that allows these groups an opportunity to expand (Stecher et al., 2013). The composition of the mucosa-associated microbiota is known to generally be distinct from the fecal microbiota (reviewed in Donaldson et al., 2015) and localized microbiota at sites of inflammation may thus not be identified in fecal samples. While fecal samples are not a suitable reflection of the specific microbiota in various intestinal compartments, they are still considered to be valuable as an alternative to invasive and potentially dangerous endoscopic procedures (Vermeire et al., 2006). The large consistency between indicator OTUs identified in the cecum and colon flush samples and the fecal samples indicates that murine stool microbial communities can indeed be diagnostic for alterations in the intestinal microbiota and is therefore a viable source for non-invasive monitoring of health-associated biomarkers. Differences between lumen and stool indicator analysis can be attributed to differences in the treatments being tested (one, two, or three cycles of DSS in the intestinal flush versus three cycles of DSS in the stool), the inclusion of the cecal microbiota in the intestinal flush samples, and the greater number of fecal pellet samples. As an alternative quantitative method to confirm selected sequencing results, we analyzed the dynamics of several target organisms in fecal samples using quantitative FISH. FISH analysis confirmed that in the feces of DSS-treated mice there was increased *Enterobacteriaceae* and *Akkermansia* sp., which have previously been identified to be increased in acute-phase colitis (Berry et al., 2012; Supplementary Figure S6). *Akkermansia* abundance peaked in the second cycle at day 39, which was also reflected by an approximately twofold enrichment in the sequencing data (untreated 6.0 ± 4.2%, DSS: 13.7 ± 6.2%; mean ± SEM). Additionally, *Bacteroides acidifaciens*, a colitis-associated species and an abundant member of the *Bacteroidaceae* family (Berry et al., 2012), also exhibited elevated levels during inflammation but then decreased rapidly post-colitis and was therefore not identified as an indicator of inflammation history. The DSS-naïve (health) indicator *Lachnospiraceae* OTU 207, which was also identified as an indicator in a previous study (called OTU 11021 in the previous study; Berry et al., 2012), was present in one DSS-naïve mouse at very high abundance and recovered after the first treatment, but was lost and did not recover again after subsequent treatments. The loss of this *Lachnospiraceae* OTU is an example of the individualized response and the eroded resilience of the murine gut microbiota after multiple DSS-induced disturbances.

## Conclusion

In the present study, using intestinal flush and stool samplings, we have characterized the response of the intestinal microbiota to repeated acute colitis episodes. Importantly, signatures in the microbiota reflected the history of colitis. The microbiota therefore represents a sensitive read-out for acute and historical health state. Using longitudinal fecal sampling of animals, we found that the microbiota of mice with a history of colitis was destabilized and exhibited a directional trajectory increasingly diverging away from the microbiota prior to treatment. OTUs associated with prior colitis episodes were identified both in intestinal flush and stool samples, and were largely consistent. Reduced community resilience may have implications for microbiome function as well as susceptibility to additional colitis events. Possible biomarkers in the human microbiota will very likely be different from those in mice, and therefore future work will be needed to establish the relevance in the context of human disease and to identify biomarkers in the human microbiota. However, our results indicate that the use of stool samples to monitor bacterial biomarkers of inflammation state in individuals with chronic and relapsing IBDs is a promising approach.

## Author Contributions

DB, OK, IR, SH, CS, and EH performed the experiments. DB, AL, MW, TD, MM, BS, CS, TU, and LK were involved in experimental design and supervision. SH and LK performed histological analysis. DB and AL analyzed sequence and FISH data and prepared the manuscript and figures. All authors reviewed the manuscript.

## Conflict of Interest Statement

The authors declare that the research was conducted in the absence of any commercial or financial relationships that could be construed as a potential conflict of interest.
