# Supplementary material for: Intestinal Microbiota Signatures Associated with Inflammation History in Mice Experiencing Recurring Colitis
Source: Front Microbiol. 2015 Dec 15;6:1408. doi: 10.3389/fmicb.2015.01408 (PMC4678223; doi:10.3389/fmicb.2015.01408)
Supplement: Supplementary file 1 [file Table_1.PDF]

**Supplementary information for:**

# Intestinal microbiota signatures associated with inflammation history in mice experiencing recurring colitis

David Berry, Orest Kuzyk, Isabella Rauch, Susanne Heider, Clarissa Schwab, Eva Hainzl, Thomas Decker, Mathias Müller, Birgit Strobl, Christa Schleper, Tim Urich, Michael Wagner, Lukas Kenner, Alexander Loy

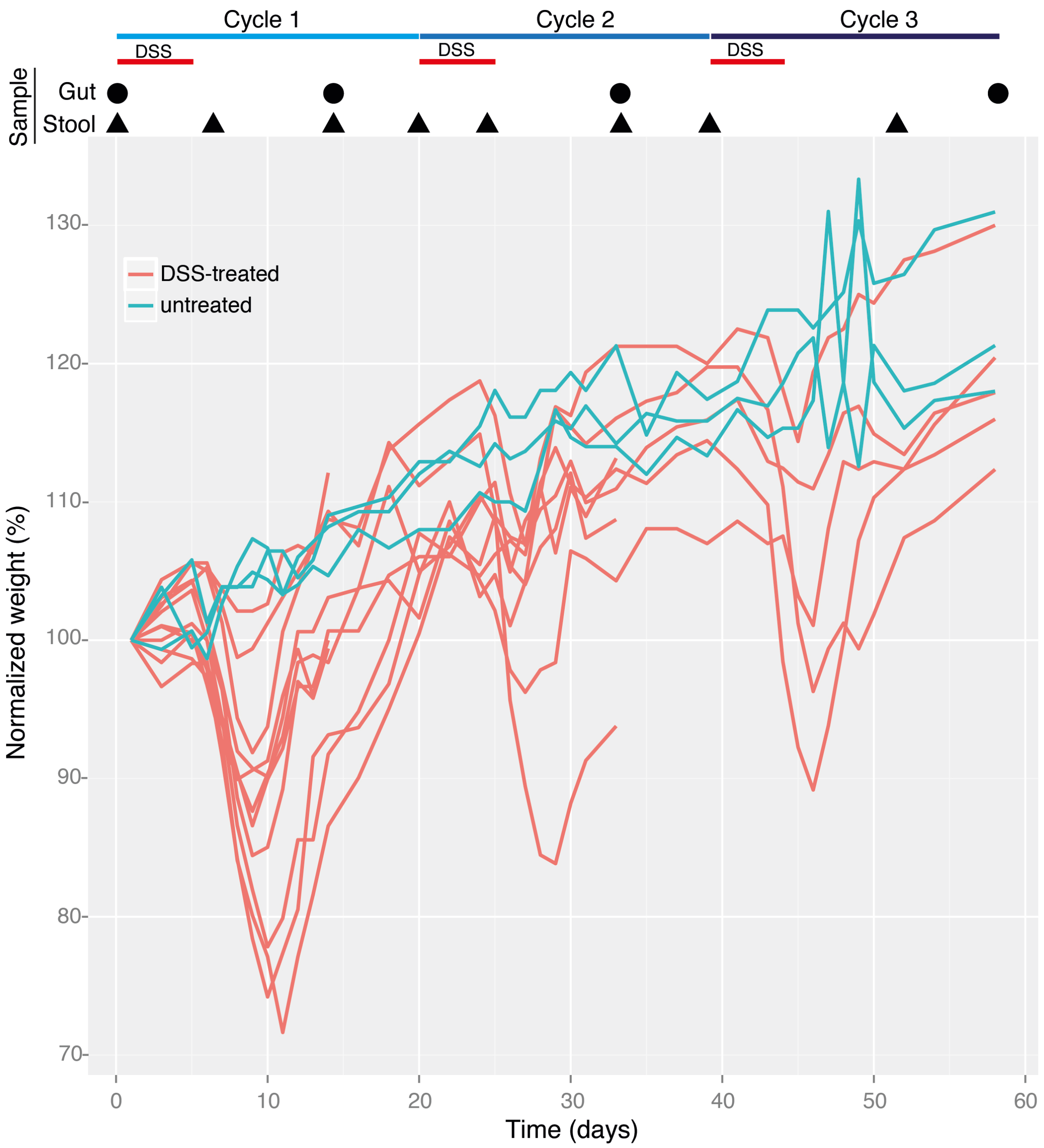


**Figure S1. Per-mouse body weight dynamics over the course of the experiment.** The change in weight (in percent) of each mouse is shown. Weight dynamics of mice given three cycles of 2% DSS in drinking water (indicated by red horizontal bars) are shown in red and weight dynamics of untreated control mice are shown in blue. Weight lines terminate upon sacrifice of mice. Mice were sampled for intestinal (cecum and colon) contents and pathology analysis as well as for stool analysis at selected time points (indicated by a circle or triangle, respectively)

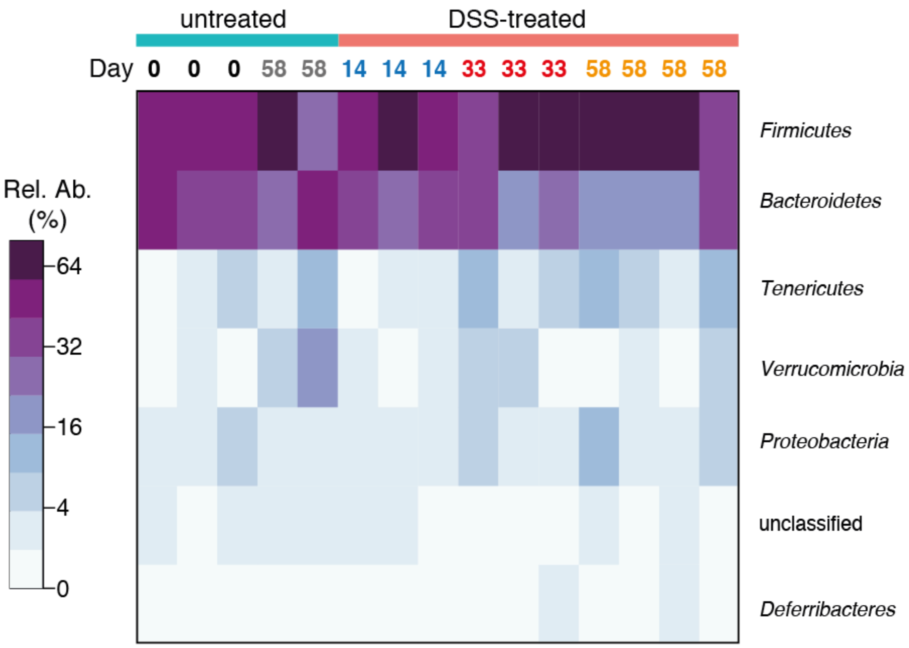


**Figure S2. Phylum-level composition of lumen flush samples.** A heatmap is shown of the relative abundance of each taxon (rows) in each sample (columns). The abundance is shown in relative percent and the scale is square root transformed to allow visualization of less abundant groups. Taxa with at least 1% relative abundance in at least one sample are shown.

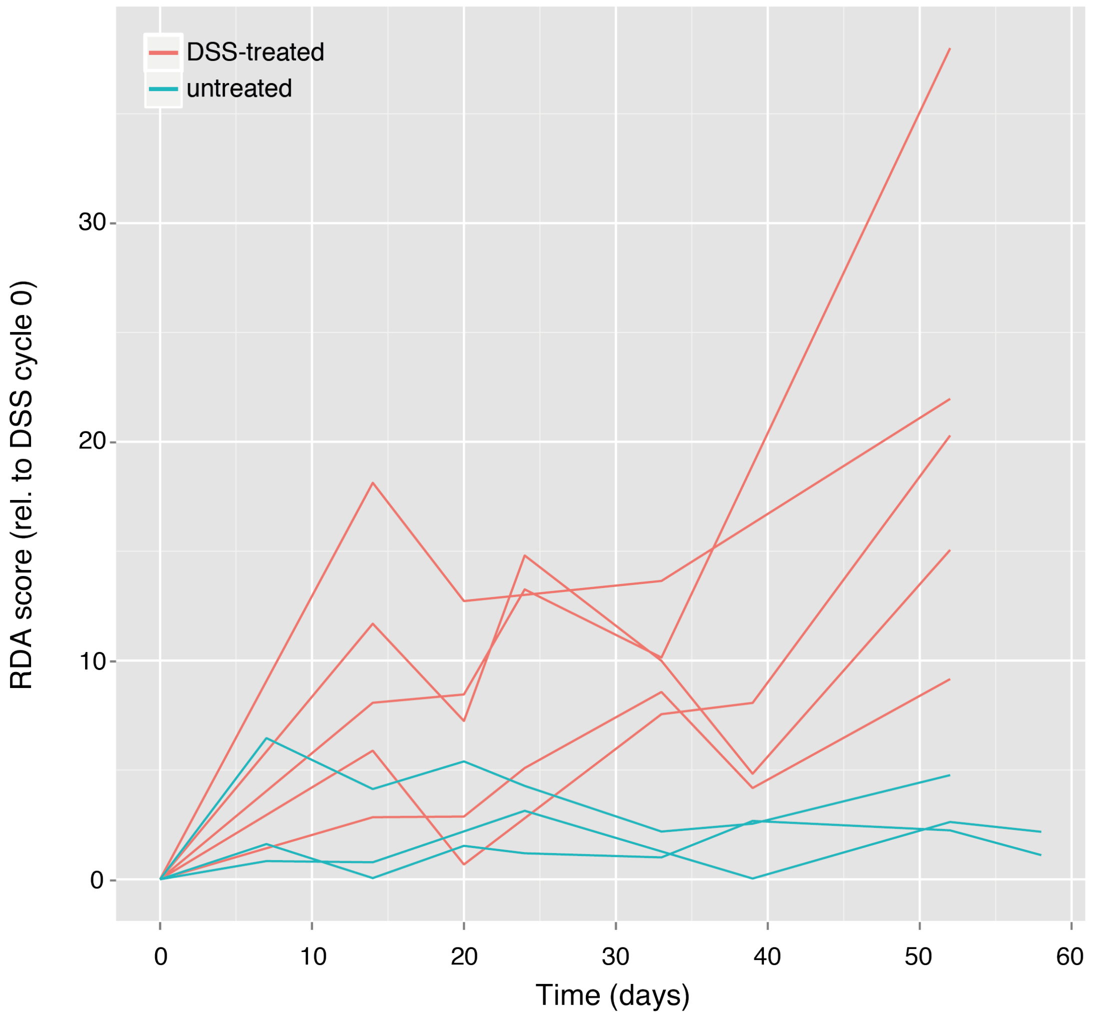


**Figure S3.** The redundancy analysis score of the microbial community at successive time points relative to day 0 for each mouse is shown, indicating divergence due to number of DSS treatments. Higher values indicate increasing divergence from the starting community. Mean values for samples from DSS-treated and untreated mice are shown in Fig. 2C.

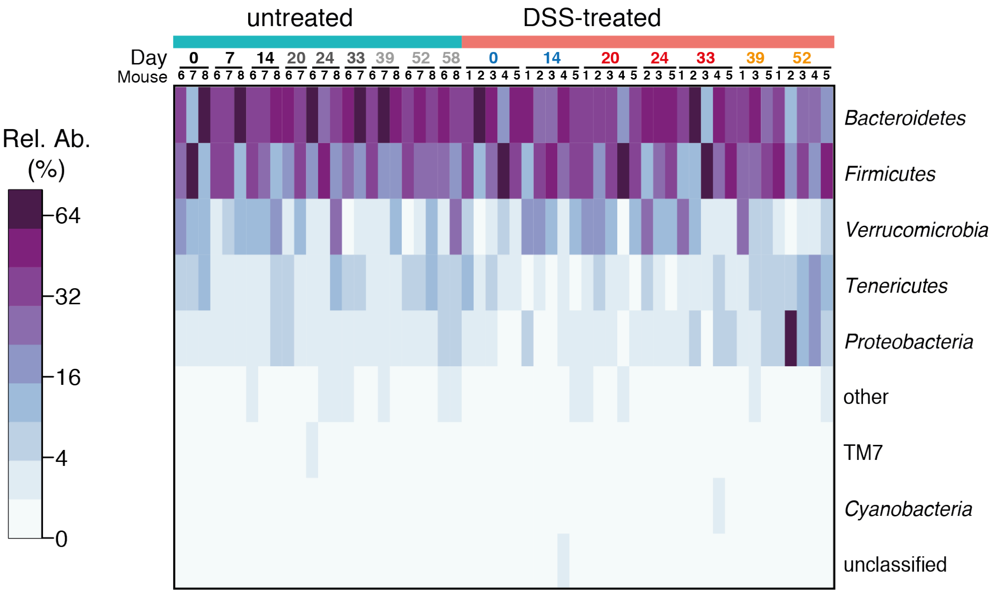


**Figure S4. Phylum-level composition of fecal pellet samples.** A heatmap is shown of the relative abundance of each taxon (rows) in each sample (columns). The abundance is shown in relative percent and the scale is square root transformed to allow visualization of less abundant groups. Taxa with at least 1% relative abundance in at least one sample are shown.

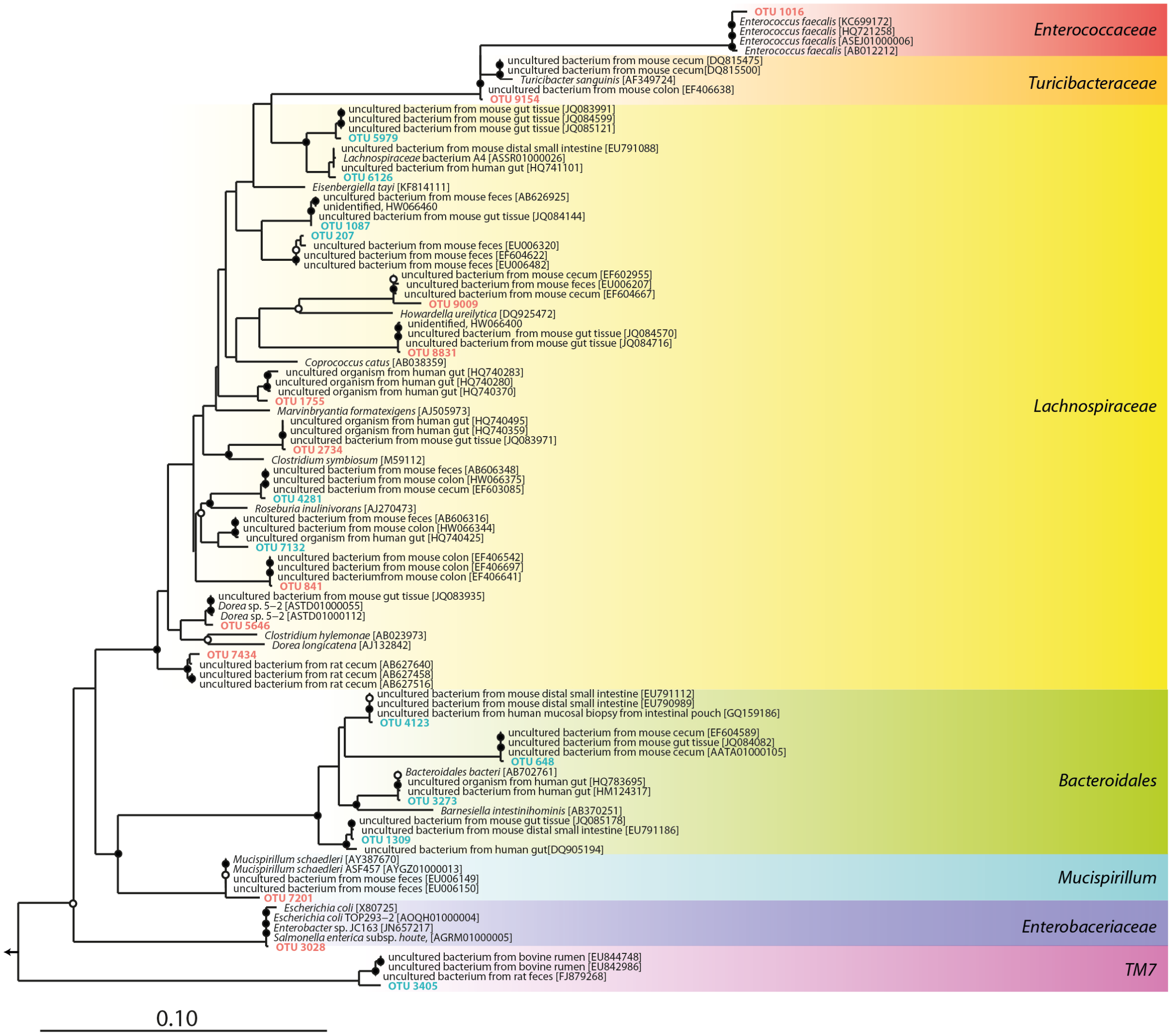


**Figure S5. Phylogenetic analysis of partial 16S rRNA gene sequences of indicator OTUs and related sequences.** The tree was produced using near-full-length sequences from organisms closely related to indicator OTU sequences (indicated in black) using RAxML with 500 bootstrap re-samplings (black circles indicate >90% support, white circles >75% support). OTU sequences were then added to the tree using the quick-add parsimony method in ARB. Indicators for prior DSS treatment and no previous treatment are shown in red and blue, respectively. The taxonomic classification of sequences is indicated. Scale bar indicates number of substitutions per site.

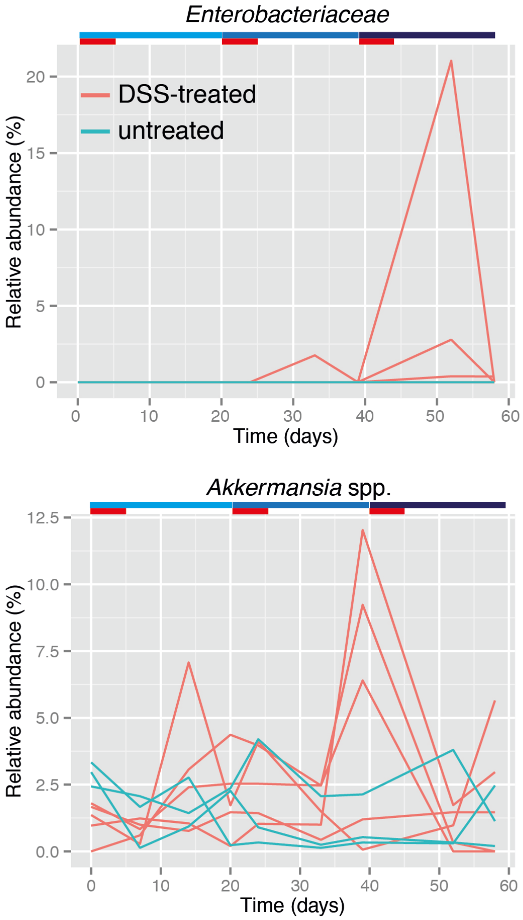


**Figure S6. Quantitative fluorescence *in situ* hybridization of target bacteria in fecal samples.** The relative abundance, represented as percent bacterial biovolume (a percentage of all microbes detected with the EUB338 probe set), of target groups is shown for each sample. Relative abundances are shown for 5 DSS-treated mice in red and 3 untreated mice in blue (absence of a line indicates that the target was not detected with FISH). Colored rectangles at the top of each graph indicate DSS treatment periods (in red) and the duration of each treatment and recovery cycle (shades of blue).

**Table S1. 16S rRNA and 23S rRNA-targeted probes used in this study**. For *Bacteroides acidifaciens* both probes are used together to cover the entire species. For *Lachnospiraceae* OTU 207, only the overlapping signal of two probes labeled with different dyes was used to ensure specificity.

| Target | Name | Sequence (5'-3') | Formamide (%) | Reference |
|---|---|---|---|---|
| All *Bacteria* | EUB338 (S-D-Bact-0338-a-A-18) | GCT GCC TCC CGT AGG AGT | 30 | (Amann et al 1990) |
| | EUB338-II (S-*-BactP-0338-a-A-18) | GCA GCC ACC CGT AGG TGT | 30 | (Daims et al 1999) |
| | EUB338 III (S-*-BactV-0338-a-A-18) | GCT GCC ACC CGT AGG TGT | 30 | (Daims et al 1999) |
| Control probe complementary to EUB338 | NON-EUB | ACT CCT ACG GGA GGC AGC | 30 | (Wallner et al 1993) |
| *Akkermansia* spp. | AKK1437 (S-G-Akk-1437-a-A-20) | CCT TGC GGT TGG CTT CAG AT | 30 | (Derrien *et al.*, 2008) |
| *Bacteroides acidifaciens* | BAC9164a-1000 (S-S-Bac-1000-a-A-23) | AAC ATG TTT CCA CAT TAT TCA GG | 30 | (Berry *et al.*, 2013) |
| | BAC9164b-177 (S-S-Bac-177-a-A-23) | CAT GCG GTA GGA CTA TGA CAT CG | 30 | (Berry *et al.*, 2013) |
| *Lachnospiraceae* OTU 207 (OTU 11021 in previous study) | LSPotu11021-1127 (S-*-Lsp-1127-a-A-20) | TTC CCA TCT TTC TTG CTG GC | 30 | (Berry *et al.*, 2012) |
| | LSPotu11021-1448 (S-*-Lsp-1448-a-A-18) | GCA GCT CCC TCC TCT CGG | 30 | (Berry *et al.*, 2012) |
| *Enterobacteriaceae* | EBAC1790 (S-*-EBAC-1790-a-A-18) | CGT GTT TGC ACA GTG CTG | 40 | (Bohnert *et al.*, 2000) |
